# Biological Intelligence Inspired Trajectory Design for Energy Harvesting UAV Networks †

**DOI:** 10.3390/s23020863

**Published:** 2023-01-11

**Authors:** Xuanlin Liu, Sihua Wang, Changchuan Yin

**Affiliations:** 1 Beijing Laboratory of Advanced Information Network, Beijing University of Posts and Telecommunications, Beijing 100876, China; 2 Beijing Key Laboratory of Network System Architecture and Convergence, Beijing University of Posts and Telecommunications, Beijing 100876, China; 3 State Key Laboratory of Networking and Switching Technology, Beijing University of Posts and Telecommunications, Beijing 100876, China

**Keywords:** unmanned aerial vehicles, wireless communications, trajectory design, foraging theory

## Abstract

In this paper, the problem of trajectory design for energy harvesting unmanned aerial vehicles (UAVs) is studied. In the considered model, the UAV acts as a moving base station to serve the ground users, while collecting energy from the charging stations located at the center of a user group. For this purpose, the UAV must be examined and repaired regularly. In consequence, it is necessary to optimize the trajectory design of the UAV while jointly considering the maintenance costs, the reward of serving users, the energy management, and the user service time. To capture the relationship among these factors, we first model the completion of service and the harvested energy as the reward, and the energy consumption during the deployment as the cost. Then, the *deployment profitability* is defined as the ratio of the reward to the cost of the UAV trajectory. Based on this definition, the trajectory design problem is formulated as an optimization problem whose goal is to maximize the deployment profitability of the UAV. To solve this problem, a foraging-based algorithm is proposed to find the optimal trajectory so as to maximize the deployment profitability and minimize the average user service time. The proposed algorithm can find the optimal trajectory for the UAV with low time complexity at the level of polynomial. Fundamental analysis shows that the proposed algorithm achieves the maximal deployment profitability. Simulation results show that, compared to Q-learning algorithm, the proposed algorithm effectively reduces the operation time and the average user service time while achieving the maximal deployment profitability.

## 1. Introduction

### 1.1. Background and Motivation

Taking advantage of their mobility and low cost, unmanned aerial vehicles (UAVs) can provide more swift deployment and better communication channels for next generation wireless communication systems [1]. In fact, UAVs have already been deployed in extensive fields [2], such as wireless power transfer, wireless sensor networks, and secure communications. However, energy limitation is still the challenge in UAV-assisted wireless networks.

Together with a couple of communication equipments consuming extra energy, the flight time of a UAV can be substantial reduced [3]. In the UAV-assisted networks, the flight time of the UAVs determines the life time of the communication networks. Optimizing the energy management of the UAVs can effectively extend the life time of the UAV-assisted networks. On the other hand, the extra payload of the assisted-UAVs increases the probability of UAV damage and malfunction [4]. This kind of risk degrades the reliability of the UAV service and affects the accuracy and performance of the UAV-assisted wireless network, which makes regular repair necessary before UAV deployment and increases the cost of maintenance.

Motivated by the aforementioned factors, we focus on a wireless network that a UAV provides service to ground users. In such a network, the UAV can harvest energy from charging stations to extend its flight time. By selecting served users and designing the trajectory, the UAV can further optimize the energy consumption. In the studied scenario, the UAV trajectory is jointly evaluated by the the maintenance costs, the energy management, the completion of users’ requests, and the user service time.

### 1.2. Related Work

The existing literature has studied a number of problems related to the energy management of UAVs for wireless communication systems, such as [5,6,7,8,9,10]. The authors in [5] derived a theoretical model on the propulsion energy consumption of UAVs, which first correlated the UAVs’ energy consumption with the varying flying speed, direction, and acceleration in UAV communications. The work in [6] investigated the energy trade-off between the communication power and the propulsion power, so as to find an energy-efficient design of UAV trajectory. In [7], the authors studied the energy-efficiency in a multi-UAV coverage deployment model by a game-theoretic framework and proposed a sub-optimal energy-efficient coverage deployment by decoupling the coverage maximization and power control. The authors in [8] studied the energy consumption and completion time trade-off in a UAV-enabled wireless power communication network, so as to achieve better communication performance. The works in [5,6,7,8] only consider the optimization of UAV energy consumption to save energy, but ignore the energy supplementary which can also extend the working time of a UAV to serve more users. The work in [9] studied the energy of solar-powered UAVs and considered the solar energy harvesting during the UAV deployment, which enhances the UAV communication capacity. The authors in [10] introduced ground solar panels to recharge UAVs and discussed the relationship between UAV battery level and UAV coverage. With the ground solar panels as supplementary, the mission duration of UAVs can be extended. However, most of the existing works such as [5,6,7,8,9,10] solves the UAV energy management problems with optimization methods, which takes too much time for UAVs to obtain the optimal policies to execute in practical environments.

A number of existing literature works [11,12,13] have studied the combination of low-complexity biological intelligence with UAV control. The work in [11] studied the collision-free trajectory problem by introducing swarm behaviors, which makes the UAV be aware of spatial-temporal constraints and eliminate collision conflicts. The authors in [12] explored the biological robustness to design a reliable multi-UAV network by adaptively resisting the node failures. In [13], the authors proposed a target searching scenario of multi-UAVs and coordinated the UAV behaviors as stigmatic and flocking behaviors. By the bio-inspired strategy, the UAVs can efficiently search and sense potential targets. Motivated by the above works [11,12,13], we model the UAV deployment as a foraging process of bacteria searching for protein to extend lifetime. In the proposed wireless network, the UAV works as a base station (BS) to serve users and searches for energy supplementary to extend the working time. In this case, the trajectory design problem of UAVs with energy management can be solved by an algorithm with low time complexity. Furthermore, the UAV deployment can be faster in practice.

### 1.3. Contributions

The main contribution of this paper is to optimize the trajectory of the UAV while jointly considering maintenance cost, the reward of serving users, the energy management during deployment, and the user service time. In this regard, our key contributions are summarized as follows:

- We propose an energy harvesting UAV network, in which the UAV can serve ground users while collecting energy from the charging stations (CSs). To serve the ground users and collect energy, the UAV must be examined and repaired before deployment. In consequence, it is necessary to jointly consider the maintenance cost, the number of users that are served by the UAV, and the energy consumption and harvesting.
- To capture the relationship among the maintenance cost, the number of users that are served by the UAV, and the energy consumption and harvesting, we model the completion of users’ data requests and the harvested energy as reward, and the energy consumption as cost. The deployment profitability is defined as the ratio of the reward achieved during the deployment to the cost of energy consumption. Given the concept of the deployment profitability, the trajectory design problem is decoupled as a decision-making problem of maximizing the deployment profitability and a queuing problem of minimizing the average user service time.
- To solve this problem, we develop a foraging-based algorithm [14]. Compared to the trajectory design algorithms such as successive convex approximation [15] and Q-learning [16,17], the proposed foraging algorithm is proved to design the UAV trajectory with the optimal deployment profitability and minimize the average service time of served users. The time complexity of the proposed algorithm is also significantly reduced to the level of polynomial.

Simulation results show that, in terms of the deployment profitability, the proposed algorithm yields up to 20.2% gain compared to the Q-learning algorithm. In terms of the average user service time, based on the optimized deployment profitability, the proposed algorithm achieves 17.3% and 8.7% reduction compared to the worst case benchmark and the Q-learning algorithm, respectively. The proposed algorithm also reduces the operation time effectively. To our best knowledge, this is the first work that uses the foraging theory to analyze the profitability of UAV deployment and design the trajectory.

### 1.4. Organization

The rest of this paper is organized as follow. The system model and problem formulation are described in Section 2. The foraging-based algorithm is introduced in Section 3. In Section 4, numerical results are presented and analyzed. Finally, conclusions are drawn in Section 5.

## 2. System Model and Problem Formulation

**Figure 1:**
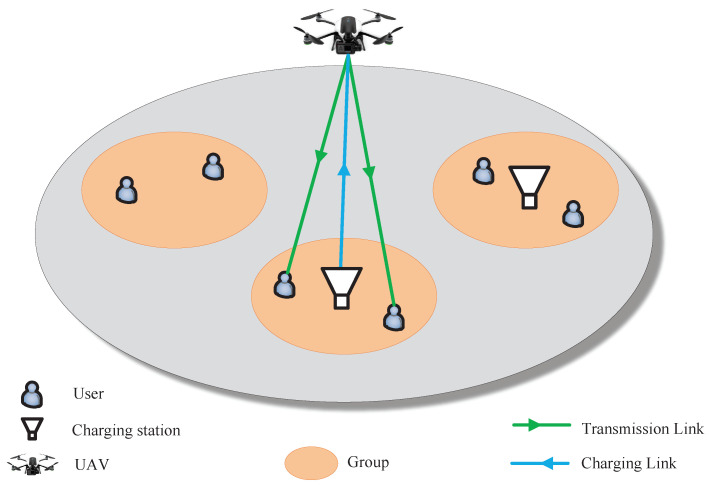
Architecture of an energy harvesting UAV network.

We consider a downlink wireless network that consists of a rotary-wing UAV and a set U of *U* users. The users are equally clustered into a set G of *G* groups, as shown in Figure 1. In these user groups, *C* groups are equipped with CSs. The CSs located at the center of user groups are made by laser transmitters so as to provide energy for the UAV installed with photovoltaic receivers by laser power. The UAV deployed at an initial position works as a BS to provide service to the users according to user’s data request $D_{i}$ and harvests energy from the CSs to extend the UAV working time. For ease of reading, we summarize the main notations in this paper in Table 1.

For each time slot τ, the UAV will serve one group of users. In particular, providing service to a group of users consists of four steps: (1) Flying to the center of group *j*, (2) Harvesting energy to charge battery if a CS exists, (3) Providing downlink transmission to complete all the data requests in a given group, and (4) Returning to the initial deployed position. Next, we first introduce the transmission model and energy consumption model of the UAV. Then, we define the deployment profitability of the UAV to evaluate the service trajectory and formulate the problem of maximizing the deployment profitability. On this basis, we further formulate the problem of minimizing the average service time of served users.

### 2.1. Transmission Model

The size of data requested by user *i* located at $\left(x_{i},y_{i}\right)$ is $D_{i}$, i∈U. After flying to the center of group *j*, whose coordinate is $\left(m_{j},n_{j}\right)$, j∈G, the UAV can first charge its battery if group *j* owns a CS. Then, the UAV BS provides service to all users in group *j* simultaneously.

The probabilistic UAV channel model is used to model the transmission link between the UAV and user *i*. Probabilistic line-of-sight (LoS) and non-line-of-sight (NLoS) links are considered in [18]. The LoS and NLoS channel gains of the UAV transmitting data to user *i* are given by [19]:

(1) $$g_{ij}^{\mathrm{LoS}}=d_{ij}^{-\alpha },$$

(2) $$g_{ij}^{\mathrm{NLoS}}=\eta d_{ij}^{-\alpha },$$

where $d_{ij}=\sqrt{{\left(x_{i}-m_{j}\right)}^{2}+{\left(y_{i}-n_{j}\right)}^{2}+H^{2}}$ is the distance between user *i* and the UAV hovering position at group *j*, *H* is the altitude of the UAV, α is the path loss exponent for the UAV transmission link, and η is an additional attenuation factor caused by the NLoS connection. The probability of the LoS link is given by [20]:

(3) $$\gamma_{ij}^{\mathrm{LoS}}=\frac{1}{1+X\exp\left(-Y\left[\phi_{ij}-X\right]\right)},$$

where *X* and *Y* are constants depended on the environment (rural, urban, dense urban, and others), $\phi_{ij}=\frac{180}{\pi }\sin^{-1}\left(\frac{H}{d_{ij}}\right)$ is the elevation angle in degree. The average channel gain from the UAV to user *i* is given by [19]:

(4) $${\bar{g}}_{ij}=\gamma_{ij}^{\mathrm{LoS}}\times g_{ij}^{\mathrm{LoS}}+\gamma_{ij}^{\mathrm{NLoS}}\times g_{ij}^{\mathrm{NLoS}},$$

where $\gamma_{ij}^{\mathrm{NLoS}}=1-\gamma_{ij}^{\mathrm{LoS}}$. Based on Shannon equation, the downlink rate of user *i* in group *j* is expressed as:

(5) $$c_{ij}=\rho_{ij}B\log_{2}\left(1+\frac{P_{ij}^{T}{\bar{g}}_{ij}}{\sigma^{2}}\right),$$

where *B* is the total bandwidth of the UAV downlink transmission, $\rho_{ij}$ is the bandwidth allocation coefficient of user *i* in group *j*, $P_{ij}^{T}$ is the transmission power of the UAV serving user *i* in group *j*, and $\sigma^{2}$ is the power of the Gaussian noise. The transmission time of each user *i* can be simply given by $t_{ij}^{T}=D_{i}/c_{ij}$ and the transmission time of group *j* is defined as the maximal transmission time of users in this group, which is given by:

(6) $$t_{j}^{T}=\max_{i\in U_{j}}t_{ij}^{T},$$

where $U_{j}$ is the set of users in group *j*. With the transmission model of UAV serving users, we can further define the energy consumption model.

### 2.2. Energy Consumption Model

In this model, the UAV can harvest energy from CSs. The UAV charges its battery to extend its working time via uplink wireless power transfer (WPT) [21]. The CS in group *j* transmits energy with the power of $P_{j}^{E}$, where $P_{j}^{E}=0$ implies that group *j* does not have a CS. Since the CSs are located at the centers of user groups and the UAV also hovers over the centers of user groups, we assume that the WPT channel is LoS-dominated so that the free-space path loss model is adopted. The path loss of the power transferred from the CS to the UAV is expressed as $h=\beta_{0}H^{-2}$, where $\beta_{0}$ denotes the power path loss at a reference distance. Hence, the received power by the UAV from CS in group *j* is given by:

(7) $$P_{j}^{C}=hP_{j}^{E}.$$

The charging time in group *j* is defined as $t_{j}^{C}$. Obviously, the UAV cannot harvest energy while serving user group *j* without a CS, which implies the charging time is $t_{j}^{C}=0$. In consequence, the energy that is harvested by the UAV from a CS in group *j* is given by:

(8) $$E_{j}^{C}=P_{j}^{C}t_{j}^{C}.$$

The CSs are the only sources that the UAV can charge the battery, so the total harvested energy of the UAV serving users in group *j* is given by $E_{j}^{+}=E_{j}^{C}$.

The energy consumption of the UAV consists of two components: (1) energy consumption of UAV-user communication and (2) energy consumption of UAV movement. The energy consumption of UAV-user communication refers to the energy that the UAV uses to complete users’ data requests. The energy consumption of UAV movement consists of the propulsion energy that the UAV takes round-trip between the initial position and group centers, and the hovering energy supporting the UAV to provide service. Next, we formulate the model of the energy consumption of the UAV.

(1) Communication Energy: The transmission power and time of the UAV serving user *i* in group *j* are defined in the previous subsection by $P_{ij}^{T}$ and $t_{ij}^{T}$, respectively. Thus, the energy consumption of the UAV transmitting data to the users in group *j* is given by:

(9) $$E_{j}^{T}=\sum_{i\in U_{j}}P_{ij}^{T}t_{ij}^{T}.$$

(2) Movement Energy: To serve a group of users, the UAV needs to fly to the center of group *j*, hover while charging and serving, and return to the initial position eventually. We assume that the horizontal velocity of the UAV is a constant *v* during the movement. The one-way propulsion energy consumption from the initial position to the center of group *j* is given by [5]:

(10) $$E_{j}^{M}=\frac{d_{j}}{v}\left(c_{1}v^{3}+\frac{c_{2}}{v}\right),$$

where $d_{j}$ represents the distance from the initial position to group *j*, $c_{1}$ and $c_{2}$ are the propulsion parameters related to the weight, wing area, and air density of the UAV. Similarly, the energy consumption of UAV moving from the center of group *j* to the initial position is $E_{j}^{M}$.

The energy consumption of UAV hovering at group *j* is given by:

(11) $$E_{j}^{H}=P^{H}\left(t_{j}^{C}+t_{j}^{T}\right),$$

where $P^{H}$ is the hovering power that depends on the UAV weight, air density, and rotor disc area [22]. For the groups without CSs, the energy consumption of UAV hovering is $E_{j}^{H}=P^{H}t_{j}^{T}$, since the UAV will not spend time to harvest energy.

In consequence, the total energy that the UAV consumes for serving a group of users is given by:

(12) $$E_{j}^{-}=E_{j}^{T}+2E_{j}^{M}+E_{j}^{H}.$$

From (12), we can see that the transmission energy consumption, the propulsion energy consumption of the round-trip, and the hovering energy consumption are jointly considered.

### 2.3. Problem Formulation

Next, we first introduce the notion of the deployment profitability and formulate the problem of maximizing the deployment profitability. Then, we formulate the problem of minimizing the average service time of served users on the basis of maximal deployment profitability.

For the service provider, deploying a UAV to serve the users in a certain area has a maintenance cost for examining and repairing the UAV, which is denoted by *Q*. The deployment profitability is used to capture the relationship among the maintenance cost, the number of users that are served, and the energy consumption and harvesting, which is given by:

(13) $$f\left(o\right)=\frac{-Q+\sum_{j\in G}o_{j}\left(q^{S}U_{j}+q^{C}E_{j}^{+}\right)}{\sum_{j\in G}o_{j}q^{C}E_{j}^{-}},$$

where $o=\left[o_{1},\ldots ,o_{j},\ldots ,o_{G}\right]$ denotes the potential served groups, $U_{j}=\left|U_{j}\right|$ is the number of users in group *j* that served by the UAV, |·| is the operator that counts the elements in a set. In particular, $o_{j}=1$ implies that the users of group *j* will be served by the UAV. Otherwise, we have $o_{j}=0$. $q^{S}$ is the income that the UAV gains by completing one user request. $q^{C}$ is the energy price per *Joule*. $q^{S}U_{j}$ represents the reward that the UAV earns by serving users in group *j*. $q^{C}E_{j}^{+}$ implies the reward that the UAV achieves by harvesting energy from the CS of group *j*. $q^{C}E_{j}^{-}$ reveals the energy cost for serving group *j*.

Having introduced the notation of deployment profitability in (13), the maximization problem can be formulated as:

(14) $$\begin{array}{cc} \max_{o,P_{ij}^{T},\rho_{ij}} & f\left(o\right), \end{array}$$

(15) $$\begin{array}{cc} s.\;t. & o_{j}\in \left\{0,1\right\}, \end{array}$$

(16) $$\begin{array}{cc} \; & \sum_{i\in U_{j}}\rho_{ij}\leq 1,\forall o_{j}=1, \end{array}$$

(17) $$\begin{array}{cc} \; & \sum_{i\in U_{j}}P_{ij}^{T}\leq P^{T},\forall o_{j}=1, \end{array}$$

where constraint (15) means $o_{j}$ is the indicator of potential served group, constraints (16) and (17) indicate that the sum of allocated bandwidth and transmission power cannot exceed the total bandwidth *B* and the UAV transmission power $P^{T}$, for potential served groups. (14) aims to select the potential served groups so that the UAV can achieve the maximal deployment profitability in any trajectory.

With the optimal group selection $o^{*}$, we further define the total service delay [17] of each user so as to design the optimal trajectory for minimizing the average service time of served users.

For user *i* in group *j* that selected by (14), the total service delay does not only include the transmission delay, it also includes the time for waiting the UAV to complete the former services. We define the UAV trajectory as $e=\left[e_{1},\ldots ,e_{\tau },\ldots ,e_{T}\right]$, where $e_{\tau }=j$ indicates that the UAV flies to group *j* at time slot τ, $T=\left|\right|o^{*}{\left|\right|}_{0}$ is the number of time slots that the UAV completes the deployment with maximal profitability, $\left|\right|\cdot {\left|\right|}_{0}$ is the 0-norm operator that counts the non-zero elements in a vector. Since the UAV serves one user group at one time slot, the number of potential served groups can be also represented by *T*. Given the UAV trajectory e, we use $t_{\tau }^{F}\left(e\right)$ to indicate the total time that the UAV returns to the initial position after serving group $e_{\tau }$. Obviously, the total time of each time slot τ can be derived by the total time of the previous time slot τ−1, which is given by:

(18) $$t_{\tau }^{F}\left(e\right)=t_{\tau -1}^{F}\left(e\right)+2\frac{d_{e_{\tau }}}{v}+t_{e_{\tau }}^{C}+t_{e_{\tau }}^{T},$$

where $t_{0}^{F}\left(e\right)=0$ represents the UAV is first deployed in the serving area. Thus, for user *i* in group *j* served in time slot τ, the total service time can be expressed by:

(19) $$t_{ij}^{W}\left(e\right)=t_{\tau }^{F}\left(e\right)+\frac{d_{j}}{v}+t_{j}^{C}+t_{ij}^{T},$$

which includes the waiting time that the UAV completes the former services, the flight time that the UAV moves from the initial position to the center of group *j*, the transmission time of completing user *i*’s request, and the charging time of the UAV if a CS is in group *j*. On this basis, we can further formulate the problem of minimizing the average user service time as:

(20) $$\begin{array}{cc} \min_{e} & \frac{\sum_{j\in o^{*}}\sum_{i\in U_{j}}t_{ij}^{W}\left(e\right)}{\sum_{j\in o^{*}}U_{j}}, \end{array}$$

(21) $$\begin{array}{cc} s.\;t. & j\in e,\forall o_{j}^{*}=1, \end{array}$$

where constraint (21) ensures that all the potential user groups can be served with the UAV trajectory e. Thus, by using (20), we can design the optimal trajectory with minimized average user service time based on the optimal served groups with the maximized deployment profitability.

Finding the optimal served groups in (14) needs to evaluate all possible permutations of group selection o. Using conventional optimization methods may not be practical for a future wireless network that consists of a large number of wireless devices, it is necessary to introduce a low complexity algorithm to find the optimal group selection and design the UAV trajectory.

## 3. Foraging-Based Trajectory Design Algorithm

To solve the deployment profitability maximization problem in (14) and the average user service time minimization problem in (20), we propose an algorithm based on foraging theory [14]. Compared to existed algorithms for UAV trajectory design [17] such as Q-learning and double Q-learning, whose operation time is based on the number of actions and states of each agent, the foraging-based algorithm can result in the maximum of the deployment profitability with a polynomial time complexity. With the maximal deployment profitability, we can further design the UAV trajectory.

The proposed foraging-based algorithm can be divided into four parts: (a) calculating the reward of serving users, the energy consumption and harvesting for each group *j*; (b) ranking the ratio of reward to cost of serving each group *j*; (c) choosing potential served groups to achieve the maximal deployment profitability; and (d) designing the trajectory for minimizing the average user service time.

Next, we first introduce the components of the proposed foraging algorithm. Then, we explain how to use the proposed foraging-based algorithm to find the optimal trajectory for the UAV so as to maximize the deployment profitability and minimize the average service time of served users. Then, we analyze the time complexity of the foraging-based algorithm.

### 3.1. Components of Foraging-Based Algorithm

In (14), maximizing the deployment profitability can be treated as a decision-making problem. In particular, to solve this problem, we must select the user groups that the UAV will serve, which can be determined by the foraging theory. The components of the foraging theory can be corresponded to this problem as follows [14]:

(1) Forager: Given the defined system model, the UAV takes actions of selecting the potential served groups and designing the trajectory. During the serving process, the UAV can be regarded as the forager.

(2) Advantage-to-disadvantage function: The optimal behavior of a forager is to maximize the generic advantage-to-disadvantage (A2D) function. In the proposed problem, the behavior of the forager UAV is to maximize the deployment profitability which depends on the potential served groups. To solve the maximization problem (14), we need to reconstruct the deployment profitability (13) into the form of A2D function. The A2D function of serving group *j* is given by:

(22) $$f\left(o\right)=\frac{-Q+\sum_{j\in G}o_{j}M_{j}}{\sum_{j\in G}o_{j}N_{j}},$$

where $M_{j}=q^{S}U_{j}+q^{C}E_{j}^{+}$ represents the reward after serving group *j*, $N_{j}=q^{C}E_{j}^{-}$ represents the energy consumption of serving the users in group *j*. (22) can also be written by:

(23) $$f\left(o\right)=\frac{o_{j^{\prime }}M_{j^{\prime }}+{\hat{M}}_{j^{\prime }}}{o_{j^{\prime }}N_{j^{\prime }}+{\hat{N}}_{j^{\prime }}},$$

where ${\hat{M}}_{j^{\prime }}=\sum_{j\in G,j\neq j^{\prime }}o_{j}M_{j}-Q$ represents the reward that the UAV serves all the groups except group $j^{\prime }$, ${\hat{N}}_{j^{\prime }}=\sum_{j\in G,j\neq j^{\prime }}o_{j}N_{j}$ indicates the cost of energy consumption of serving all the groups except group $j^{\prime }$.

(3) Profitability of objects: The forager makes decisions based on the profitability of its objects. In the maximization problem (14), the objects that the forager UAV aims at are the user groups. For group *j*, the reward that the UAV can gain stems from providing service and harvesting energy. The cost is the energy consumption of providing service to the users in each group. Thus, the profitability of group *j* can be defined as $p_{j}=M_{j}/N_{j}$. Similarly, ${\hat{p}}_{j}={\hat{M}}_{j}/{\hat{N}}_{j}$ can be regarded as the alternative profitability of group *j*, which is the deployment profitability resulting from serving all the groups except group *j*. In the following subsection, we will introduce the use of the profitability and the alternative profitability for the foraging-based algorithm to find the potential served groups and achieve the maximal deployment profitability.

### 3.2. Implementation of Foraging-Based Algorithm

In the studied model, we formulate the problem as: (1) maximizing the deployment profitability, and (2) minimizing the average service time of served users. From (14), we can see that the optimization variables are the group selection indicator o, the power allocation $P_{ij}^{T}$, and the bandwidth allocation coefficient $\rho_{ij}$. In particular, the served group selection depends on the result of power and bandwidth allocation. By optimizing the resource allocation of each group, the forager UAV can obtain the group information including the service time, energy consumption of each group. We can also decouple the maximization problem into two parts: (a) resource allocation for each user groups, and (b) group selection for achieving the maximal deployment profitability. The optimization of resource allocation is widely studied in existing literature using convex optimization [23] or reinforcement learning [24] methods. In this work, we mainly focus on the foraging-based group selection. Thus, the maximization problem (14) can be simplified as:

(24) $$\begin{array}{cc} \max_{o} & f\left(o\right), \end{array}$$

(25) $$\begin{array}{cc} s.\;t. & o_{j}\in \left\{0,1\right\}, \end{array}$$

where the resource allocation of power and bandwidth has already been solved by existing methods.

With the optimal resource allocation, the deployment profitability can be treated as a linear function of a vector o. To obtain the maximum of the deployment profitability, we need to differentiate $f\left(o\right)$ with respect to $o_{j^{\prime }}$. Thus, we temporarily adopt the served indicator relaxation, where $o_{j^{\prime }}$ can be any real value in $\left[0,1\right]$, so as to make the function $f\left(o\right)$ continuous in its domain. Later, we will show that the optimal solution of the served indicator $o_{j^{\prime }}$ must be either 1 or 0 even though the feasible domain of $o_{j^{\prime }}$ is relaxed. The partial differential of $f\left(o\right)$ with respect to $o_{j^{\prime }}$ is given as follow:

(26) $$\frac{\partial f}{\partial o_{j^{\prime }}}=\frac{M_{j^{\prime }}{\hat{N}}_{j^{\prime }}-N_{j^{\prime }}{\hat{M}}_{j^{\prime }}}{{\left(o_{j^{\prime }}N_{j^{\prime }}+{\hat{N}}_{j^{\prime }}\right)}^{2}}.$$

From (26), it is noted that if $M_{j^{\prime }}{\hat{N}}_{j^{\prime }}-N_{j^{\prime }}{\hat{M}}_{j^{\prime }}$ is negative, then $f\left(o\right)$ is maximized by choosing the smallest $o_{j}^{\prime }$. Alternatively, if $M_{j^{\prime }}{\hat{N}}_{j^{\prime }}-N_{j^{\prime }}{\hat{M}}_{j^{\prime }}$ is positive, then $f\left(o\right)$ is maximized by choosing the largest $o_{j}^{\prime }$. Thus, we can obtain a policy for the UAV selecting the user groups:

(27) $$o_{j^{\prime }}=\left\{\begin{array}{cc} 0, & \mathrm{if}p_{j^{\prime }}\leq {\hat{p}}_{j^{\prime }},\\ 1, & \mathrm{if}p_{j^{\prime }}>{\hat{p}}_{j^{\prime }}. \end{array}\right.$$

From (27), we can see that the UAV will select group $j^{\prime }$ to serve the users once the profitability of group $j^{\prime }$ is larger than the alternative profitability of group $j^{\prime }$. $p_{j^{\prime }}$ can be easily obtained based on the reward and cost of group $j^{\prime }$ itself. However, calculating ${\hat{p}}_{j^{\prime }}$ needs to know the reward and cost of all other groups, which takes up a large amount of calculation.

In consequence, we introduce a sorting algorithm to solve the simplified problem (24) by only calculating profitability $p_{j}$ of each group. According to their profitability, the groups are descending ranked as $p_{s_{1}}>p_{s_{2}}>\ldots >p_{s_{G}}$, where $s_{k}=j$ indicates the profitability of group *j* is the *k*-th largest of all the groups. The group selection o is default 0. Starting from the most profitable group, the UAV decides to serve the first *k* groups to increase the deployment profitability, until the termination condition is satisfied. To find the termination condition of group selection, we present the following result.

**Theorem** **1.** The deployment profitability $f\left(o^{*}\right)$ achieves maximum when the termination condition is satisfied, which is given by:

(28) $$f\left(o^{*}\right)=\frac{-Q+\sum_{o^{*}}o_{j}^{*}M_{o_{j}^{*}}}{\sum_{o^{*}}o_{j}^{*}N_{o_{j}^{*}}}>p_{s_{k+1}}.$$

**Proof.**  Please refer to Appendix A.    □

From Theorem 1, we can see that the UAV selects each potential served group with the largest group profitability until the deployment profitability of the current served group selection $o^{*}$ is larger than the group profitability of the (k+1) largest group. In other words, the current group selection $o^{*}$ makes the deployment profitability $f\left(o^{*}\right)$ greater than the profitability of any unselected group. Based on the above procedure, the foraging-based group selection algorithm for maximizing the deployment profitability performed by the UAV is summarized in Algorithm 1.

With the optimal group selection $o^{*}$, we can design the UAV trajectory so as to minimize the average service time of served users. From (19), we can see that the waiting time of each served user consists the fixed part and the variable part, which can be expressed by:

(29) tijWe=abtτFe⏟variable+abdjv+tjC+tijT⏟fixed.

**Algorithm** **1** Foraging-based group selection1:**Input:** User positions $\left(x_{i},y_{i}\right)$, user requests $D_{i}$, group locations $\left(m_{j},n_{j}\right)$, and charging power $P_{j}^{C}$2:**Init:** UAV position, group selection o=03:Optimize the power allocation $P_{ij}^{T}$ and bandwidth allocation coefficient $\rho_{ij}$ for each group and each user4:Calculate the profitability $p_{j}=M_{j}/N_{j}$ for each group5:Rank the profitability from large to small6:**repeat**7:   Select the next potential served group $j^{\prime }$ from the remaining set with the largest profitability8:   $o_{j^{\prime }}\leftarrow 1$9:   Delete group $j^{\prime }$ from the group set G10:**until** Satisfy (28)11:$o^{*}\leftarrow o$12:**Output:** Optimal group selection $o^{*}$ with maximal deployment profitability

For any trajectory e, the fixed part of user service time cannot be optimized. In this case, the minimization problem (20) can be simplified as minimizing the average total time of all the time slot τ, which is given by:

(30) $$\begin{array}{cc} \min_{e} & \frac{1}{T}\sum_{\tau \in e}t_{\tau }^{F}\left(e\right), \end{array}$$

(31) $$\begin{array}{cc} s.\;t. & j\in e,\forall o_{j}^{*}=1, \end{array}$$

where $U_{j}$ can be reduced since the users are equally clustered in groups. The simplified minimization problem (30) can be treated as a queuing problem of the potential served groups where completing the service of group *j* spends a time of ${\hat{t}}_{j}=2\frac{d_{j}}{v}+t_{j}^{C}+t_{j}^{T}$. It is easy to know that the group with the shortest service time ${\hat{t}}_{\min}$ should be first served so as to achieve the average total service time. In this case, we rank the service time of all the potential served groups selected by $o^{*}$ in ascending order as ${\hat{t}}_{z_{1}}<{\hat{t}}_{z_{2}}<\ldots <{\hat{t}}_{z_{T}}$, where $z_{k}=j$ indicates the service time of group *j* is the *k*-th shortest of all the potential served groups. The UAV trajectory can be written as:

(32) $$e=\left[z_{1},z_{2},\ldots ,z_{T}\right],o_{z_{k}}^{*}=1.$$

Based on the above procedure, the trajectory design algorithm for minimizing the average service time of all served users is summarized in Algorithm 2.
**Algorithm** **2** Trajectory design for minimizing the average service time1:**Input:** Optimal served group selection $o^{*}$2:**Init:** UAV trajectory e, time slot τ=03:Calculate the service time ${\hat{t}}_{j}$ of each potential served group *j*4:Rank the service time from small to large5:**repeat**6:   τ←τ+17:   Select the next served group $j^{\prime }$ with the τ-th shortest service time8:   $e\leftarrow \left[e,j^{\prime }\right]$9:**until**τ=T10:$e^{*}\leftarrow e$11:**Output:** Optimal UAV trajectory $e^{*}$ with minimal average user service time

### 3.3. Complexity of Foraging-Based Algorithm

In the studied problem, the resource allocation subproblem is first solved by existing methods. The proposed algorithm mainly focuses on the group selection and trajectory design parts based on an optimized resource allocation policy. In these two parts, the intermediate variables can be easily obtained by the algebra calculation, the time complexity of ergodic calculation is at a linear level of $O\left(G\right)$. The complexity of ranking the profitability is based on the chosen sorting algorithm. In the proposed algorithm, we use the *QuickSort* to rank the profitability and the service time. The time complexity of *QuickSort* is $O\left(n\log_{2}n\right)$ [25], where *n* is the number of the elements to be sorted. In particular, the proposed algorithm first sorts the profitability of all *G* groups to select potential served groups. After that, the proposed algorithm ranks the service time of *T* potential served groups to design the UAV trajectory. In general, the total time complexity of the proposed algorithm can be regarded as $O\left(G\log_{2}G+T\log_{2}T\right)$, which is related to the number of total groups and potential served groups. Compared to those machine learning algorithms [26] utilized in wireless communications, whose operation time depends on the network scale and learning parameters, the proposed foraging algorithm has a stable and lower theoretical complexity.

## 4. Simulation Results

In our simulations, we consider a circular wireless network with a radius R=200 m, in which a rotary-wing UAV is deployed to serve users and harvest energy. The UAV keeps an altitude H=100 m and a horizontal speed v=30 m/s during movement. The initial position of the UAV BS is set to the origin $\left(0,0\right)$. *G* groups with a radius $R_{G}=20$ m are uniformly distributed in the network and each group *j* is with $U_{j}=3$ users. Half of the groups are equipped with CSs, *C* is equal to the integer part of G/2. For implementing and verifying the proposed foraging-based algorithm, we use the Matlab tools for simulation. Unless state otherwise, the parameters we used during the simulations are listed in Table 2. The functionality of the proposed algorithm can be divided into two parts: (1) group selection, and (2) trajectory design. For the group selection part, the optimization of resource allocation is pre-processed by methods in [23] and will not be discussed in the following results. We compare the deployment profitability with Q-learning algorithm in [17]. For the trajectory design part, we first generate the optimal group selection policy with the maximal deployment profitability by the foraging-based algorithm. Then, while completing the service of selected groups, we compare the average total service time with the worst case scenario and the Q-learning algorithm. In our simulations, the worst case scenario indicates that the UAV trajectory leads to the longest average total service time. All the statistical results are averaged over 500 independent runs.

**Figure 2:**
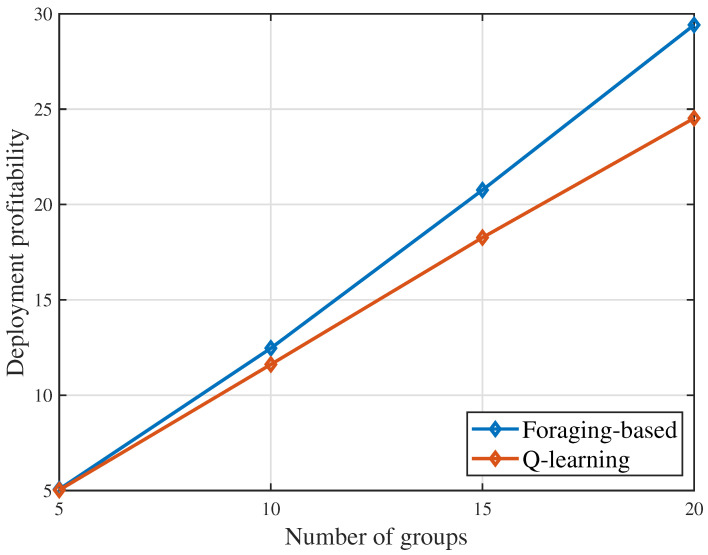
Deployment profitability as the number of groups varies.

Figure 2 shows that the deployment profitability changes as the number of groups changes. From Figure 2, we can see that, for both considered algorithms, the deployment profitability increases with the number of groups increasing. This is due to the fact that as the number of user groups increases, the user groups that can be served by the UAV and the energy that is harvested by the UAV increase. In Figure 2, when the number of groups G=20, the foraging-based algorithm achieves a deployment profitability of 29.42, while the Q-learning algorithm achieves a deployment profitability of 24.52. The proposed foraging-based algorithm yields up to 20.0% gain in terms of deployment profitability compared to the Q-learning algorithm. This gain stems from the fact that the proposed algorithm can find the optimal potential served groups which maximizes the deployment profitability, while the Q-learning algorithm may find a sub-optimal group selection which leads to a worse value.

**Figure 3:**
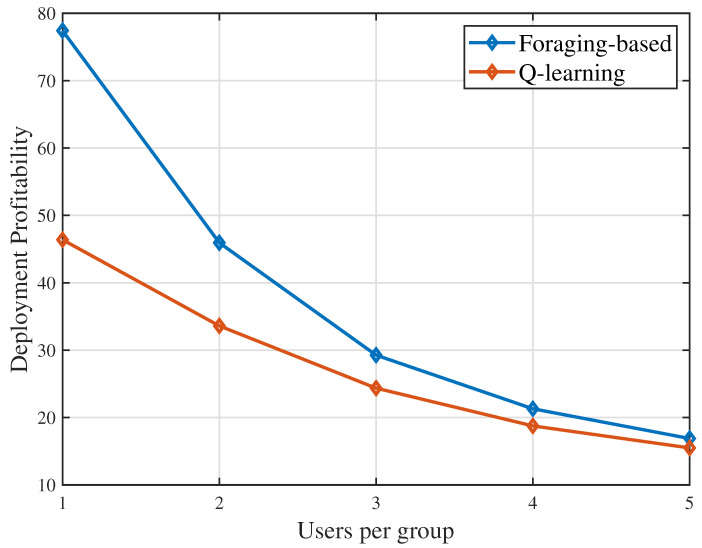
Deployment profitability as the users per group vary.

Figure 3 shows that the deployment profitability changes as the number of users in a group changes. In Figure 3, we can see that both considered algorithms achieve lower deployment profitability with the number of users per group increasing. This is due to the fact that, for each group, the increasing of users also leads to the increasing of required data. The UAV needs to hover longer so as to complete the user requests and consumes more hovering energy. Increased energy consumption leads to the reduction of the deployment profitability.

In Table 3, we show that the operation time of algorithm changes as the number of groups changes. The operation time records how fast the algorithm can select potential served users. In our simulation, the Q-learning converges after around 2000 iterations. From Table 3, we can see that, the operation time of Q-learning algorithm is more than ten thousand times larger than that of foraging-based algorithm. This is due to the fact that the foraging-based algorithm provides a solution to the proposed maximization problem with the time complexity of $O\left(G\log_{2}G+T\log_{2}T\right)$. Compared to the Q-learning, whose operation time depends on the number of iterations, the foraging-based algorithm effectively shortens the time that the UAV spends on potential served group selection. Table 3 also shows that the operation time of both algorithms increases as the number of groups increases. For the proposed foraging-based algorithm, having more groups increases the elements to sort, which takes up the major operation time of the proposed algorithm. For Q-learning algorithm, having more groups increases the number of actions to implement, which extends the steps of each iteration.

**Figure 4:**
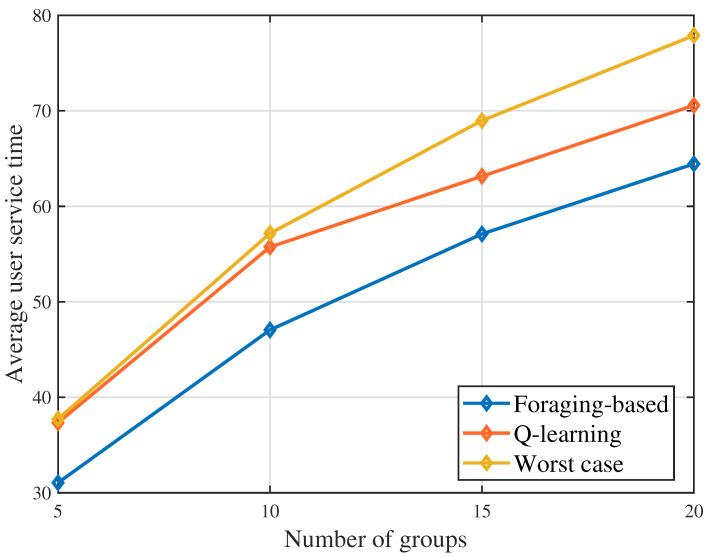
Average user service time as the number of groups varies.

Figure 4 shows that the average user service time changes as the number of groups varies. With the increasing of groups, the average user service time also increases. This is due to the face that the increasing of groups gives the UAV more options of selecting potential served groups so as to increase the deployment profitability. Serving more groups makes the users in the later served groups have to wait for a longer time before the UAV comes and provides service. From this figure, we can also see that the proposed foraging-based algorithm achieves lower results than Q-learning algorithm. This is because the proposed algorithm designs the UAV trajectory based on a greedy policy, which solves the queuing problem of minimizing the queuing time. However, the optimization process of the Q-learning algorithm may stuck in a sub-optimal trajectory. In Figure 4, when the number of groups G=20, in terms of the average user service time, the foraging-based algorithm achieves up to 17.3% and 8.7% reduction compared to the worst case baseline and the Q-learning algorithm, respectively.

**Figure 5:**
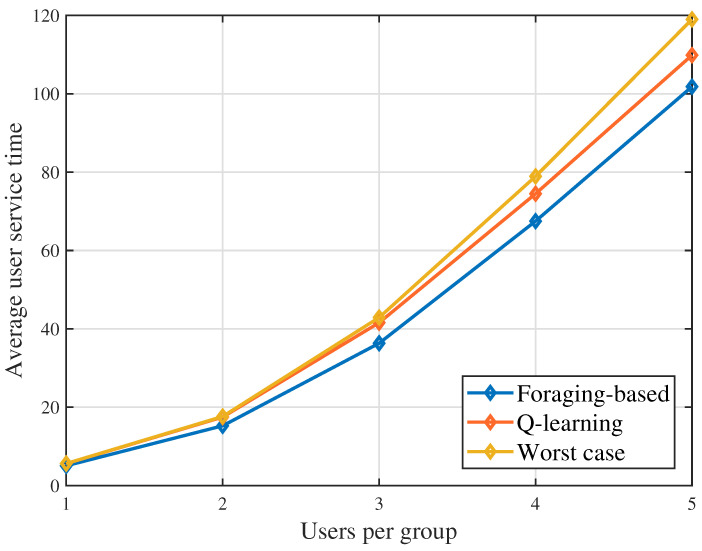
Average user service time as the users per group vary.

In Figure 5, we show that the average user service time changes as the users in a group varies. The average user service time increases when more users are in a group. This is because the UAV needs to spend longer time to complete the user requests when serving a group. In this case, the service time of each group increases. The users in the later served group have to spend more time waiting the UAV completes the data transmission in the previous groups.

**Figure 6:**
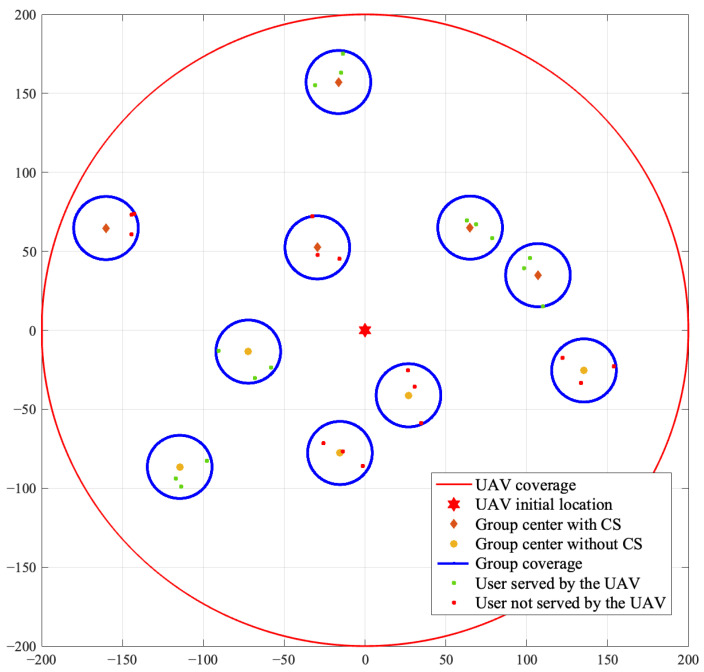
An illustrative example of the users served following the UAV trajectory designed by the proposed algorithm.

Figure 6 shows the users served by the UAV of an arbitrary case after the UAV follows the optimal trajectory designed by the foraging-based algorithm. In this case, G=10 groups are distributed in the wireless network and the UAV selects the potential served users with the maximal deployment profitability. From Figure 6, we can see that the UAV does not only serve the groups with CSs and the groups near to the initial position, but also serves the groups without CSs and the groups far from the initial position. This is due to the fact that the UAV jointly considers the distance and the existence of CSs, which decides the consumed energy and the harvested energy, and further affects the profitability of serving a group.

## 5. Conclusions

In this paper, we have developed a novel framework to evaluate the deployment profitability for the UAV. The UAV can gain reward by serving users in groups and harvesting energy from CSs. The cost of the UAV consists of the consumed energy during transmitting data and movement. To solve this problem, we have developed a novel algorithm based on foraging theory. The proposed foraging-based algorithm enables the UAV to find the optimal trajectory that achieves the maximal deployment profitability and minimized the average service time of served users. By ranking the profitability of each group and choosing the group from the largest profitability, the UAV selects potential served groups. The UAV trajectory is further designed based on a queuing problem. Simulation results have shown that the proposed approach with much lower computational complexity yields significant performance gains of the deployment profitability compared to prior Q-learning algorithm. With the optimized deployment profitability, the proposed approach also reduces the average service time of served users.

## Figures and Tables

**Table 1:** List of notations.

Notations	Description
G	Set of user groups
U	Set of users
$U_{j}$	Set of users in group *j*
$D_{i}$	Data request of user *i*
$d_{ij}$	Transmission distance between user *i* and the UAV at group *j*
*H*	UAV altitude
${\bar{g}}_{ij}$	Average channel gain of probabilistic UAV channel
$c_{ij}$	Downlink rate of user *i* in group *j*
*B*	Bandwidth
$\rho_{ij}$	Bandwidth allocation coefficient
$P_{ij}^{T}$	Transmission power of serving user *i*
$t_{ij}^{T}$	Transmission time of user *i*
$t_{j}^{T}$	Transmission time of group *j*
$E_{j}^{C}$	Harvested energy while UAV serving group *j*
$E_{j}^{T}$	Transmission energy while UAV serving group *j*
$E_{j}^{M}$	Movement energy while UAV serving group *j*
$E_{j}^{H}$	Hovering energy while UAV serving group *j*
*Q*	Maintenance cost
o	Group selection indicator
e	UAV trajectory
$f\left(o\right)$	Deployment profitability of UAV trajectory
$t_{\tau }^{F}\left(e\right)$	Total time after UAV serving a group at time slot τ
$t_{ij}^{W}\left(e\right)$	Total service time of user *i* in group *j*
$M_{j}$	Reward of serving group *j*
$N_{j}$	Cost of serving group *j*
$p_{j}$	Profitability of group *j*

**Table 2:** System parameters.

Parameters	Description	Values
*H*	UAV altitude	100 m
α	Path loss exponent	2
η	NLoS attenuation factor	0.3
X,Y	Environment constants	11.95, 0.136
$\sigma^{2}$	Noise power	−84 dBm
*B*	Bandwidth	1 MHz
$\beta_{0}$	Channel power gain	−30 dB
$c_{1},c_{2}$	Propulsion parameters	$9.26\times 10^{4}$, 2250
$P^{T}$	Transmission power	0.2 W
*Q*	Maintenance cost	10
$q^{S}$	Service reward	2
$q^{C}$	Energy cost	$1.4\times 10^{-3}$

**Table 3:** Operation time (s) as the number of groups varies.

Number of Groups	Foraging-Based Algorithm	Q-Learning Algorithm
5	$5.8\times 10^{-5}$	0.725
10	$6.9\times 10^{-5}$	1.263
15	$1.12\times 10^{-4}$	1.779
20	$1.14\times 10^{-4}$	2436

## Data Availability

Not applicable.

## Appendix A. Proof of Theorem 1

Theorem 1 presents the termination condition (28) of UAV selecting the potential served groups so as to maximize the deployment profitability. To prove that the group selection $o^{*}$ achieves the maximal deployment profitability when the inequality holds, we use the contradiction method.

Suppose there exists an unselected group $j^{\prime }$, i.e., $o_{j^{\prime }}^{*}=0$, whose profitability $p_{j^{\prime }}$ is smaller than the deployment profitability of group selection $o^{*}$, we have:

(A1) $$p_{j^{\prime }}=\frac{M_{j^{\prime }}}{N_{j^{\prime }}}<f\left(o^{*}\right)=\frac{-Q+\sum_{o^{*}}o_{j}^{*}M_{o_{j}^{*}}}{\sum_{o^{*}}o_{j^{\prime }}^{*}N_{o_{j^{\prime }}^{*}}},$$

and a group selection o, which is defined as:

(A2) $$o_{j}=\left\{\begin{array}{cc} 1, & o_{j}^{*}=1\mathrm{or}j=j^{\prime },\\ 0, & \mathrm{otherwise}. \end{array}\right.$$

The deployment profitability $f\left(o\right)$ is larger than the maximal deployment profitability $f\left(o^{*}\right)$. The following inequality holds:

(A3) $$f\left(o\right)-f\left(o^{*}\right)=\frac{-Q+\sum_{o^{*}}o_{j}^{*}M_{o_{j}^{*}}+M_{j^{\prime }}}{\sum_{o^{*}}o_{j^{\prime }}^{*}N_{o_{j^{\prime }}^{*}}+N_{j^{\prime }}}-\frac{-Q+\sum_{o^{*}}o_{j}^{*}M_{o_{j}^{*}}}{\sum_{o^{*}}o_{j^{\prime }}^{*}N_{o_{j^{\prime }}^{*}}}.$$

Since $N_{j}$ represents the energy consumption of serving the users in group *j*, which is a positive number, both the denominators of the two fractions are positive. Thus, (A3) can be rewritten as $\sum_{o^{*}}o_{j}^{*}N_{o_{j}^{*}}M_{j^{\prime }}-\left(-Q+\sum_{o^{*}}o_{j}^{*}M_{o_{j}^{*}}\right)N_{j^{\prime }}>0.$ Then, we have:

(A4) $$p_{j^{\prime }}=\frac{M_{j^{\prime }}}{N_{j^{\prime }}}>\frac{-Q+\sum_{o^{*}}o_{j}^{*}M_{o_{j}^{*}}}{\sum_{o^{*}}o_{j}^{*}N_{o_{j}^{*}}}=f\left(o^{*}\right).$$

From (A4), we can see that the profitability of serving group $j^{\prime }$ is actually larger than the maximal deployment profitability of group selection $o^{*}$. Here, inequality (A4) contradicts to the condition in (28).

Therefore, there does not exist any group $j^{\prime }$ out of the group selection $o^{*}$, which can increase the deployment profitability $f\left(o^{*}\right)$. Thus, the optimal group selection $o^{*}$ achieves the maximal deployment profitability when the UAV selects the first *k* groups with the largest group profitability.
